# Ionic Liquid-Enhanced Assembly of Nanomaterials for Highly Stable Flexible Transparent Electrodes

**DOI:** 10.1007/s40820-024-01333-4

**Published:** 2024-03-04

**Authors:** Jianmin Yang, Li Chang, Xiqi Zhang, Ziquan Cao, Lei Jiang

**Affiliations:** 1 Key Laboratory of Bio-Inspired Materials and Interfacial Science, Technical Institute of Physics and Chemistry, Chinese Academy of Sciences, Beijing, 100190 People’s Republic of China; 2 University of Chinese Academy of Sciences, Beijing, 100049 People’s Republic of China; 3 College of Chemistry and Chemical Engineering, Chongqing University of Technology, Chongqing, 400054 People’s Republic of China; 4 Binzhou Institute of Technology, Binzhou, 256600 People’s Republic of China; 5 Nanomics Biotechnology Co., Ltd., Hangzhou, People’s Republic of China

**Keywords:** Ionic liquids, Assembly, Silver nanowires, MXene nanosheets, Flexible transparent electrodes

## Abstract

The controlled assembly of nanomaterials has demonstrated significant potential in advancing technological devices. However, achieving highly efficient and low-loss assembly technique for nanomaterials, enabling the creation of hierarchical structures with distinctive functionalities, remains a formidable challenge. Here, we present a method for nanomaterial assembly enhanced by ionic liquids, which enables the fabrication of highly stable, flexible, and transparent electrodes featuring an organized layered structure. The utilization of hydrophobic and nonvolatile ionic liquids facilitates the production of stable interfaces with water, effectively preventing the sedimentation of 1D/2D nanomaterials assembled at the interface. Furthermore, the interfacially assembled nanomaterial monolayer exhibits an alternate self-climbing behavior, enabling layer-by-layer transfer and the formation of a well-ordered MXene-wrapped silver nanowire network film. The resulting composite film not only demonstrates exceptional photoelectric performance with a sheet resistance of 9.4 Ω sq^−1^ and 93% transmittance, but also showcases remarkable environmental stability and mechanical flexibility. Particularly noteworthy is its application in transparent electromagnetic interference shielding materials and triboelectric nanogenerator devices. This research introduces an innovative approach to manufacture and tailor functional devices based on ordered nanomaterials.

**Supplementary Information:**

The online version contains supplementary material available at 10.1007/s40820-024-01333-4.

## Highlights

- We present a method that utilizes ionic liquid-enhanced nanomaterial assembly to fabricate highly stable and large-area MXene-silver nanowire electrodes with ordered layered structures.
- This approach emphasizes the use of hydrophobic and nonvolatile ionic liquids, which form stable interfaces with water by reducing interface energy, preventing the sedimentation loss of nanomaterials during assembly.
- The prepared electrodes not only exhibit excellent optoelectronic properties (9.4 Ω sq^−1^ sheet resistance and 93% transmittance), but also have exceptional antioxidant capacity.

**Supplementary Information:**

The online version contains supplementary material available at 10.1007/s40820-024-01333-4.

## Graphical abstract

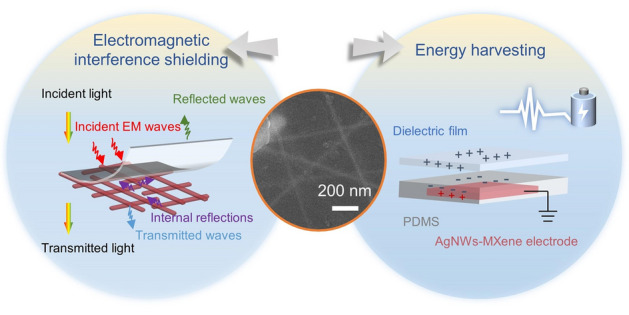

## Introduction

Nanomaterial (NM)-based flexible transparent electrodes (FTEs) have gained widespread popularity in portable and wearable electronics due to their exceptional attributes, including high transparency, low resistance, flexible, and formability. These versatile materials find applications in smart touch screens [1, 2], electroluminescent devices [3, 4], flexible displays [5, 6], sensors [7], and more. Several methods for fabricating FTEs from NMs have been reported, encompassing vacuum filtration [8], rod coating [9], spin-coating [10], spray coating [11], printing [12] and interfacial self-assembly [13, 14]. Among these approaches, interfacial self-assembly has garnered significant attention owing to its simplicity and cost-effectiveness. This technique results in NM-based FTEs with a well-ordered layered structure, leading to high optical transmittance and low sheet resistance. For example, our group employed interfacial self-assembly assisted by poly(vinyl pyrrolidone) (PVP) to fabricate a well-ordered layered graphene film with 80% transmittance [15]. Lee et al. developed a straightforward self-assembly method to produce aligned metal grids from metal nanoparticles, achieving a transmittance of over 73% [16]. Yu et al. utilized a Langmuir − Blodge strategy to manipulate nanowire assemblies for ordered layered FTEs, resulting in an 86% transmittance [4]. Wei et al. introduced a simple waterbath pulling method to align the silver nanowires (AgNWs) into an ordered array structure, achieving an 86.3% transmittance [17]. However, these nanomaterials assembled at the water–air interface are prone to sinking due to their own gravity, leading in both low assembly efficiency and high material loss.

In addition, how to transfer the assembled nanomaterials to the target substrate surface in an efficient, cost-effective, and non-destructive way is also needs to be carefully considered. Currently, some reported transfer methods including pulling [17] and scooping [18] are slow and inevitably generates losses during the transfer process, leading to the destruction of the assembled nanomaterial film. However, the self-climbing process, proposed in our previous works [15, 19], is spontaneous and guided by the difference in surface tension between the wetted substrate surface and the assembled NM film. This transfer way exhibits favored characteristics of fast, non-destructive, and autonomous transfers, providing an optimal transfer strategy for the preparation of high-performance NM-based FTEs.

As for widely reported NM-based FTEs, graphene-based FTEs have encountered limitations due to their relatively low electrical conductivity [20]. Metal meshes, while possessing high electrical conductivity, can introduce Moiré fringes due to the interaction between overlaid repetitive structures, which can be detrimental to device performance [21]. In contrast, AgNW-based FTEs, prepared through self-assembly methods, exhibit an ordered and layered arrangement structure, resulting in low sheet resistance and high optical transmittance [22]. However, the instability of AgNWs, given their susceptibility to oxidation, cannot be ignored. To enhance their stability, various secondary conductive materials have been considered, including ionogels [8, 23, 24], metal oxides [25], graphene [26], and poly(3,4-ethylenedioxythiophene): poly(styrene-sulfonate) (PEDOT:PSS) [27]. Unfortunately, the use of ionogels often reduces the conductivity of AgNW electrodes. Metal oxides and graphene are typically deposited through vapor deposition-based methods, resulting in high process costs. Additionally, the coating of PEDOT: PSS may cause corrosion of the AgNWs due to its poor environmental stability. In light of these challenges, Mxene [28], a new class of two-dimensional (2D) transition-metal carbides and nitrides, has garnered increasing attention. MXene offers favorable metallic conductivity, an ultrathin layer structure, active surfaces, and high stability [29], making it a suitable protective coating for AgNWs to guard against damage from air exposure. Consequently, the combination of MXene nanosheets and AgNW networks presents a promising solution for the preparation of highly stable FTEs [30–32]. However, achieving highly stable and large-area AgNW-based FTEs with an ordered layered structure through a highly efficient and low-loss self-assembly method remains a significant challenge.

Herein, we present an innovative method that utilizes ionic liquid (IL) enhancement in the assembly of nanomaterials combining with self-climbing process to fabricate highly stable flexible transparent electrodes with a well-ordered layered structure. The introduction of hydrophobic and nonvolatile ILs creates stable interfaces with water. This stability, in turn, enhances the assembly of NMs at the IL-water interface. This advancement enables the large-scale production of a 20 cm-wide roll of AgNW-MXene composite film capable of stably illuminating a blue LED. The resulting composite film, characterized by its well-ordered layered structure, boasts a remarkable transmittance of 93% and a low sheet resistance of approximately 9.4 Ω sq^−1^. Furthermore, thanks to the MXene nanosheet coating, the composite film demonstrates exceptional stability. It remains stable both in ambient air at room temperature for 60 days and in challenging environments, including exposure to high temperatures of up to 200 °C and immersion in a Na_2_S solution for 1 h. The high-performance composite film exhibits an outstanding electromagnetic interference shielding effectiveness (EMI SE) of 30.1 dB, surpassing the industry-standard requirement of 20 dB [33]. Furthermore, we constructed a triboelectric nanogenerator (TENG) device based on the composite film. This TENG can generate a peak voltage of 70 V, showcasing its potential for powering electronic watches or time meters.

## Experimental Section

### Materials

MAX phase Ti_3_AlC_2_ powder (200 mesh size sieve, 98%) were purchased from Beijing Lianlixin Technology Co., Ltd, China. Lithium fluoride (LiF, AR) was purchased from Aladin. Hydrochloric acid (HCl, 36% ~ 38%) was purchased from Sigma-Aldrich. AgNWs (5 mg mL^−1^ in ethanol with an average length of ~ 50 μm and diameter of ~ 30 nm) were purchased from Zhejiang Kechuang Advanced Materials. IL of 1-ethyl-3-methylimidazolium bis(trifluoromethylsulfonyl)imide ([EMIm][NTf_2_]) was purchased from Lanzhou Yulu Fine Chemical Co., Ltd. (GanSu, China) and have a purity > 99%. PVP was purchased from Tokyo Chemical Industry Co., Ltd. Poly(ethylene terephthalate) (PET) (200 μm in thickness) was purchased from Kaivo Optoelectronic Technology Co., Ltd. (Zhuhai, China). N-methylpyrrolidone (NMP) was purchased from Sigma-Aldrich. Ethanol, sodium chloride and toluene were purchased from Aladdin. All materials were used as obtained unless otherwise indicated.

### Preparation of Ti_3_C_2_T_x_ MXene Nanosheets, AgNW-MXene Composite Electrodes and Transparent AM-TENG

#### Preparation of Ti_3_C_2_T_x_ MXene Nanosheets

Ti_3_C_2_T_x_ MXene nanosheet was synthesized by etching MAX phase Ti_3_AlC_2_ with LiF/HCl as previously reported [34]. Typically, 1 g LiF and 20 mL HCl (9 M) were mixed by stirring in a 50 mL Teflon vessel, after which 1 g Ti_3_AlC_2_ powder was slowly added into the mixture and the reaction was then heated at 35 °C for 24 h in an oil bath under stirring. After the reaction, the acidic product was repeatedly washed with deionized water by centrifugation at 3500 rpm for 5 min several times until reached a neutral pH of ≥ 6. The homogeneous delaminated MXene supernatant was obtained by sonicating in an ice bath under Ar flow for 1 h and followed by centrifugation for 1 h at 3500 rpm. The prepared MXene supernatant was then treated by prefreezing and vacuum freeze-drying processes to get dried MXene powders. The MXene ink prepared by mixing MXene powders, PVP (10 wt%) and IL (1 vol%) in NMP solvent contains a MXene concentration of about 0.5 mg mL^–1^.

#### Interfacial Assembly and Sontaneous Climbing of AgNWs and MXene Nanosheets

The AgNW dispersion was diluted with ethanol into desired concentration of 0.5 mg mL^–1^. Then, the dispersion of AgNW with no additive, toluene additive, or IL additive (1 vol%) was injected onto the water surface in crystallizing dish in a rate of 10 mL h^–1^, forming a layer of transparent ultrathin AgNW film. In this case, AgNW dispersions with different additives were investigated to illustrate the impact of additive on the assembly of AgNWs. The precleaned PET substrate treated by plasma for 5 min was wetted by deionized water and then inserted into the water surface. The formed AgNW film at the IL − water interface could climb up spontaneously along the wetted substrate. Finally, the assembled AgNW film was transferred onto the PET surface and then dried thoroughly at room temperature. The same process was applied on the coating of MXene film. The dried AgNW-PET film was treated by plasma and then wetted by deionized water, realizing the climbing of MXene film onto the prewetted AgNW surface, forming the AgNW coated with MXene film (AgNW-MXene composite film).

#### Fabrication of Large-area Layered AgNW-MXene Composite Electrodes

The large-area AgNW-MXene electrodes with layered homogeneous structures were prepared as following: First, the prewetted PET substrate was inserted into the water surface, and then the assembled AgNW film on the water surface climbed up along the wetted substrate. The repetitions of this step enables multiple climbs of the AgNWs. Second, the obtained AgNW/PET film was immersed in a 2 M sodium chloride solution for 30 s, and then washing with deionized water and ethanol several times. Finally, the welded AgNW/PET film was wetted by water and then inserted into the surface where the MXene film was assembled, leading to the climbing of MXene film onto the surface of AgNW/PET film. At last, a 20 cm-wide roll of transparent AgNW-MXene electrode was obtained and then stored in a glove box.

#### Fabrication of Flexible and Transparent AM-TENG

The AM-TENG device was assembled by packaging the AgNW-MXene electrode between two PDMS layers. Initially, the prepolymer PDMS solution was cured at 70 °C for 2 h in a square mold. Then, a prepared AgNW-MXene film connecting a Cu wire by a silver paste was coated on the PDMS layer. The prepolymer solution of PDMS was dropped again to cover the AgNW-MXene film, curing again at 70 °C for 2 h. Finally, the AM-TENG device was fabricated with a area of 4 × 4 cm^2^.

### Characterization

The structure and morphology of AgNW-MXene films were examined by scanning electron microscope (SEM, SU8010) and atomic force microscope (AFM, Dimension FastscanBio). The contact mode was adopted in the AFM measurement. Sheet resistance of the AgNW-MXene film was measured on a four-point probe resistivity measurement system (Guangzhou 4-probe Tech Co. Ltd., RTS-9). The effective sheet resistance was obtained by averaging the sheet resistance at five different positions. Optical transmission spectra were recorded on a UV–Vis-NIR spectrophotometer (Lambda 900, PerkinElmer) with bare PET as the reference material in the range of 400–800 nm. Electrochemical measurements were carried out on an electrochemical workstation (CHI660E, Shanghai Chenhua Instruments, Inc.), using a conventional three-electrode test cell equipped with a cupping machine (LTS150/M). The EMI shielding properties of the AgNW film, welded AgNW film and welded AgNW-MXene film with a dimension of 22.58 × 10.14 mm^2^ were tested by a network vector analyzer (Agilent 5234B) in 8 − 12 GHz (X-band) based on the waveguide method. The electrical performances of the AM-TENG including output current, voltage and charge were characterized by using an electrometer (Keithley, 6514). The crystal structure of the synthesized MXene nanosheet was observed by a X-ray diffraction (XRD, D8 focus). The components of the AgNW film before and after NaCl were investigated by using the Fourier transform infrared spectroscopy (FTIR, Excalibur 3100) and X-ray photoelectron spectroscopy (XPS, ESCALAB 250Xi). The surface tensions of the interfacially assembled AgNW film and MXene film were measured in situ by using a dynamic contact angle measurement instrument (DCAT21) with a platinum plate-normal method. The Zeta-potentials of the AgNW and MXene nanosheets aqueous solutions were examined using a Zeta potential analyzer (Nano ZS).

## Results and Discussion

**Fig. 1:**
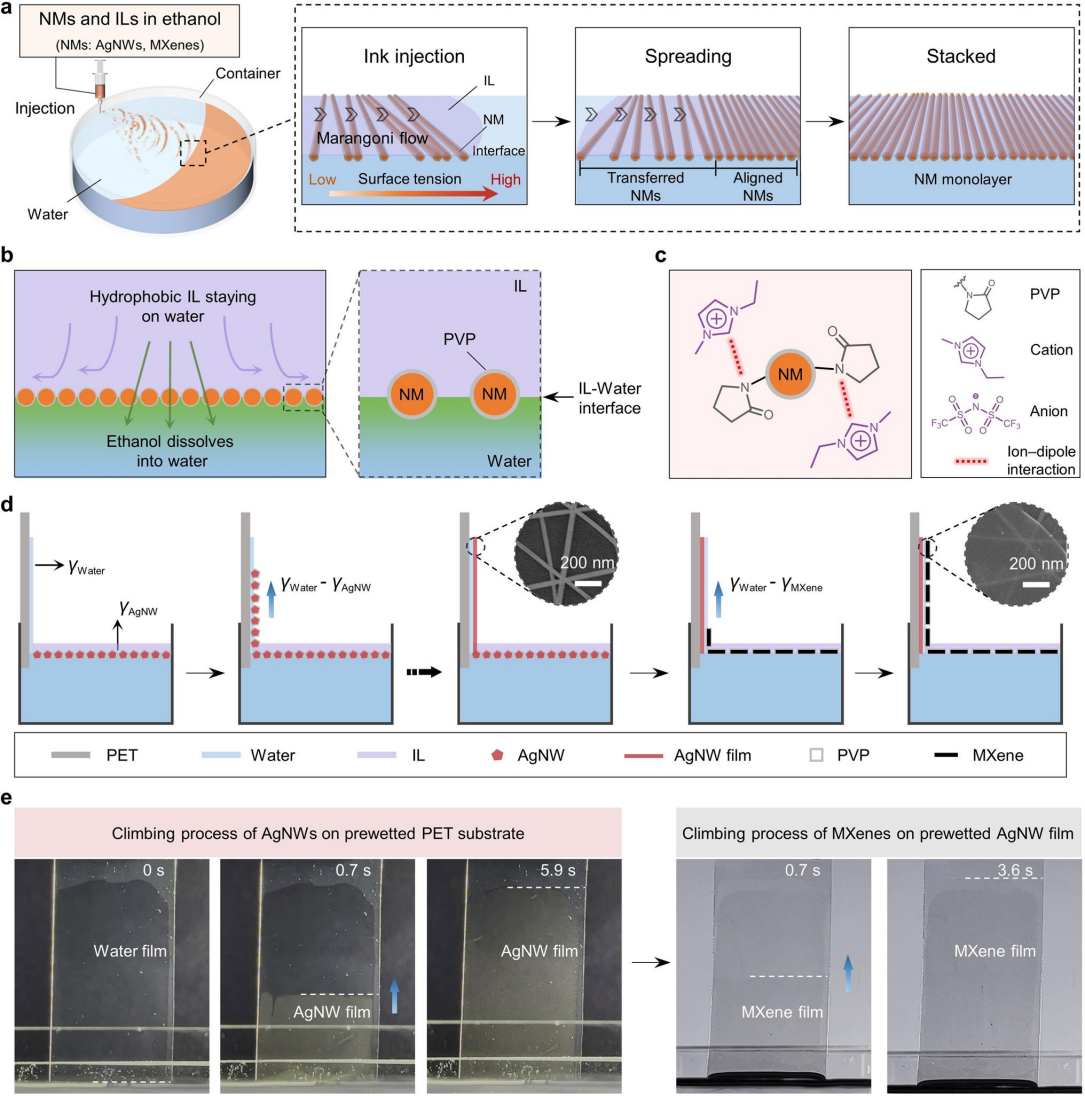
Schematic and structure of the AgNW-MXene film prepared by IL-enhanced assembly combining with wetting-induced self-climbing processes. **a** After injecting the ethanol solution containing NMs and ILs onto the surface of water, NMs rapidly disperses across the IL-water interface due to the Marangoni effect, resulting in the formation of a monolayer assembly of NMs. **b** Schematic illustrations of NMs assembly at the IL-water interface induced by the individual miscibility of each component with water and IL. **c** Molecular interaction between ILs and the PVP. **d** Illustration of surface tension (γ) difference that drives the assembled AgNW and MXene film to flow from the IL-water interface onto the prewetted PET surface. The insets are the enlarged SEM images of the AgNW film and AgNW coated with MXene film. **e** Time-lapse images of wetting-induced climbing process for the AgNW film and MXene film assembled at the IL-water interface. The dash line marks the real-time position of the climbing film

The process of the IL-enhanced assembly of NMs (*e.g.,* AgNWs and MXenes) is shown in Fig. 1a. Once the ethanol solution containing NMs and ILs touched the surface of water, water-miscible ethanol dissolved into water, whereas hydrophobic ILs stayed on the water (Fig. 1b). This formed an interface between water and IL. NMs with the amphiphilic PVP ligands settled at the IL-water interface, due to low interface energy effect. The molecular interaction between ILs and PVP further promoted the assembly of NMs at the interface (Figs. 1c and S1) [35, 36]. Dissolution of ethanol in water caused a decrease in local surface tension, which created a surface tension gradient around the solution. This gradient triggered a Marangoni flow from the near to the distant, which dragged the floating mass including the NMs and hydrophobic ILs. Since Marangoni flow carried the mass rapidly, NMs could be assembled as a monolayer. The interfacially assembled NM monolayer film can be transferred to hydrophilic PET via a robust wetting-induced climbing strategy [15]. The self-climbing is driven by the difference in surface tension between the wetted PET substrate and the assembled NM film. Wherein, the PET substrate wetted by water has a surface tension of 72.9 mN m^−1^. Representative cases using AgNWs and MXenes are demonstrated (Fig. 1d). Using a platinum plate-normal method, the surface tensions of the interfacially assembled AgNW and MXene films were measured to be 44 and 45.3 mN m^−1^, respectively (Fig. S2). Thus, the difference in surface tension between the wetted PET and AgNWs is 28.9 mN m^−1^. And the wetted PET shows a surface tension difference of 27.6 mN m^−1^ compared to MXene. The time-lapse images of self-climbing process are shown in Fig. 1e, and the sizes of the PET film and the AgNW/MXene conductive film are 50 × 25 and 40 × 25 mm^2^, respectively. Due to the notable variation in surface tension, the interfacially assembled AgNW and MXene films promptly climbed on the prewetted substrate surface at extremely fast rates of 170 and 278 mm^2^ s^–1^ (Movies S1 and S2) [19]. As a result, the prepared AgNW film and AgNW-MXene composite film exhibited highly aligned and homogeneous structures with low surface roughness, as shown in Figs. 1d and S3. Moreover, well-ordered AgNW network films with different layers were achieved by a repetitive climbing process (Fig. S4). A 20 cm-wide roll of transparent AgNW-MXene electrode on a PET substrate was obtained, which can stably light a blue LED lamp (Fig. S5 and Movie S3). Therefore, the IL-enhanced assembly of NMs combined with self-climbing processes enables the preparation of a highly stable and transparent AgNW-MXene composite film with low loss of quality.

The remarkable transparency of the AgNW-MXene composite film primarily relies on the successful fabrication of delaminated MXenes. To achieve this, MXene nanosheets were synthesized from Ti_3_AlC_2_ MAX phase powder through an etching route, as illustrated in Fig. S6a [37]. The phase transition of MXene from Ti_3_AlC_2_ precursors was examined using X-ray diffraction (XRD), as depicted in Fig. S6b. In the XRD pattern of MXene, the disappearance of the diffraction peak at the (104) planes located at 39° and the shift of the (002) peak from 9.5° to 5.8° indicate the successful removal of the Al layers through etching [30]. Furthermore, Fig. S6c presents a scanning electron microscopy (SEM) image of ultrathin MXene nanosheets, measuring 1 ~ 2 μm in diameter, deposited on an anodic aluminum oxide (AAO) substrate. These MXene nanosheets exhibit exceptional transparency. Meanwhile, atomic force microscopy (AFM) analysis (Fig. S6d, e) determined the thickness of the synthesized MXene nanosheets to be approximately 1 ~ 2 nm, confirming the acquisition of one or two layers of MXene. In addition, the zeta potential of the synthesized MXene nanosheets was found to be -33.4 mV, a significant difference from the zeta potential of AgNWs (−11.9 mV) (Fig. S7). This distinction results in a robust attractive force that enhances the mechanical durability of the AgNW-MXene composite film.

**Fig. 2:**
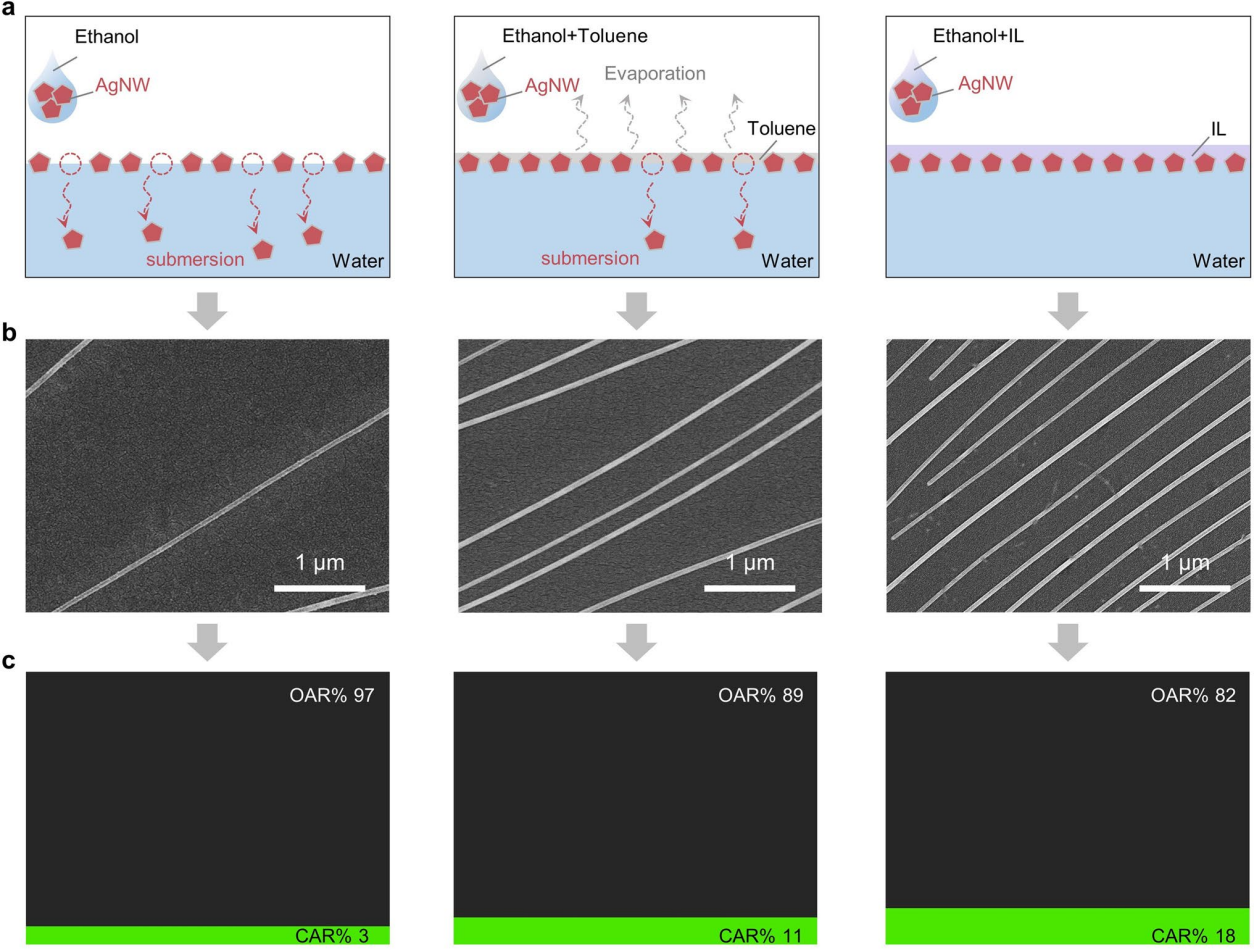
The influence of different additives on assembly of AgNWs onto water surface. **a** Schematic diagrams of AgNWs assembled on the water surface in three modes (no additive, toluene additive, and IL additive). **b** SEM images of the AgNWs assembled on the water surface with no additive, toluene additive and IL additive, respectively. **c** Corresponding images illustrating the area fraction of the open area ratio (OAR) to the covered area ratio (CAR)

To reveal the mechanism of IL-enhanced interfacial assembly of NMs, three comparative experiments (no additive, toluene additive, and IL additive) were performed. As a demonstration, AgNWs were selected as the mode of the NMs. As shown in Fig. 2a, PVP-wrapped AgNWs were assembled on the water surface once a solution droplet touched the water surface, but sinking of AgNWs occurred due to their own gravity when no additives were present. When toluene was used as an additive, it could form a toluene-water interface to stabilize the assembly of AgNWs. However, the volatile characteristics of toluene easily caused the toluene-water interface to disappear, ultimately leading to the sinking of the interface-assembled AgNWs. By contrary, hydrophobic and nonvolatile ILs could form stable interfaces with water, which avoids the sedimentation of AgNWs assembled at the interface. In other words, under the premise of the same injection concentration of AgNWs, IL additive makes the AgNWs assembled at the interface more denser, which minimizes the waste of AgNWs. As shown in Fig. 2b, when the IL additive was added, a denser AgNW film with a closely arranged structure was obtained, compared to both the no additive and toluene additive. In order to gain a better intuitive understanding of the specific density of AgNWs, the area fraction of the open area ratio (OAR) to the covered area ratio (CAR) was introduced. The OAR is the ratio of the blank area to the total area of the PET, while CAR is the ratio of the PET area occupied by the AgNWs to the total area of the PET. The relationship between the OAR and CAR is as follows: CAR + OAR = 1, which represents the total area of the PET. As shown in Fig. 2c, the CAR reached the maximum value of 18% when adding the IL additive, indicating that the more AgNWs assembled at the IL-water interface with less sinking. Therefore, the addition of IL additive enhances significantly the interfacial assembly of NWs.

**Fig. 3:**
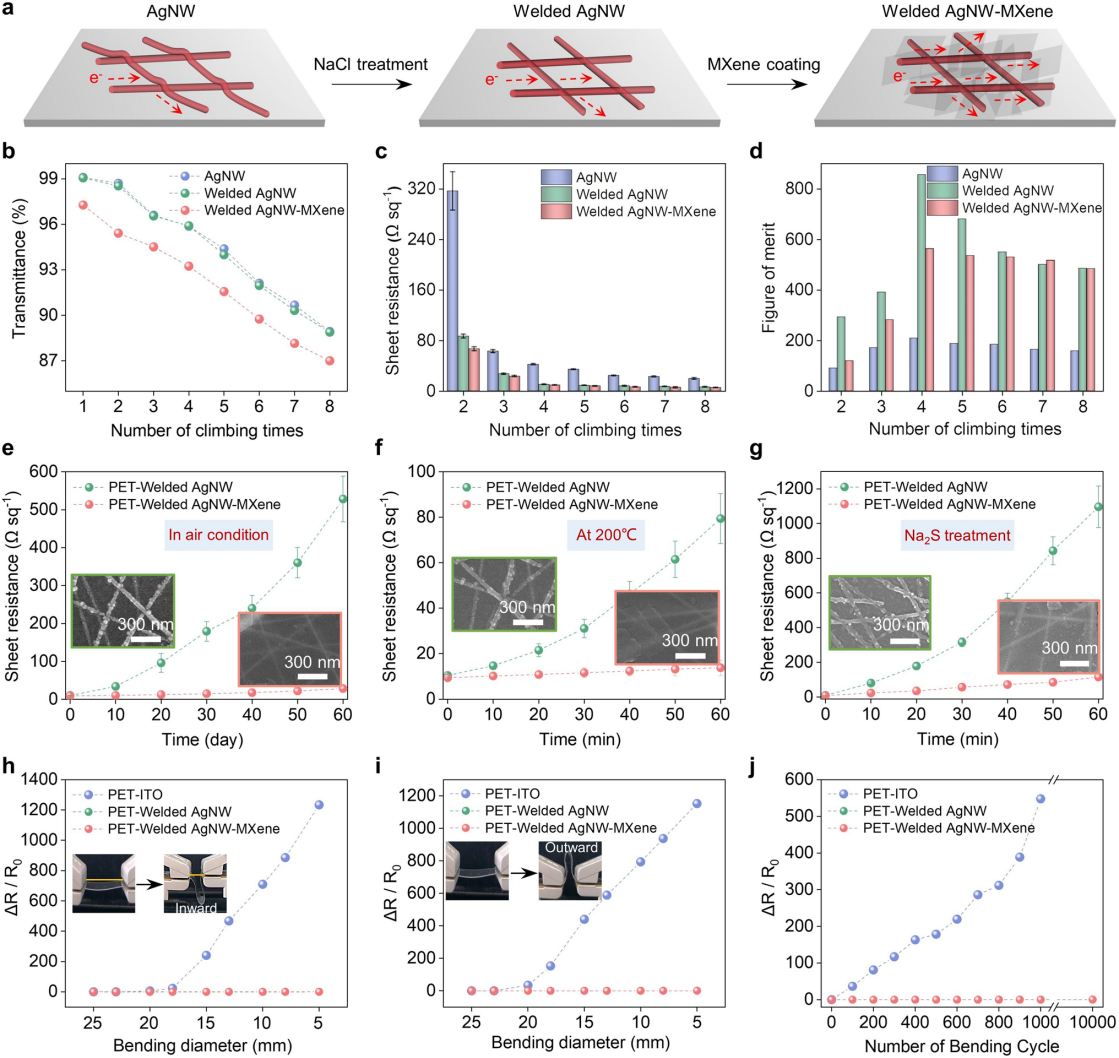
Tunable optoelectric performance and excellent mechanical property of the AgNW-based films. **a** Schematic diagrams of the electron motion of the AgNW film, welded AgNW film and welded AgNW-MXene film. The dependence of **b** transmittance, **c** sheet resistance and **d** Figure of Merit of the AgNW film, welded AgNW film and welded AgNW-MXene film on the number of AgNW climbing times. Changes in sheet resistance of the welded AgNW film and welded AgNW-MXene film **e** exposed to air at room temperature for 2 months, **f** under 200 °C for 1 h, and **g** immersed in a 5 wt% Na_2_S solution for 1 h. Insets are the SEM images of the welded AgNW film and welded AgNW-MXene film under corresponding treatments. The variation in relative sheet resistance of the ITO, welded AgNW and welded AgNW-MXene film as a function of bending inward **h** and outward **i** with a diameter from 25 mm to 5 mm. Insets are the photographs of the welded AgNW-MXene film in normal and bending state, respectively. **j** Variation in ΔR / R0 versus the number of bending outward cycles from a diameter of 25 mm to 5 mm for the ITO, welded AgNW and welded AgNW-MXene film

The fabrication of AgNW-MXene composite films from AgNW films involves two key steps: NaCl welding and MXene coating. NaCl welding aims to minimize the wire-wire junction resistance of AgNWs by eliminating PVP from their surface [38]. This removal is crucial because PVP molecules serve as encapsulants in commercial AgNW dispersions. This process is illustrated in Fig. S8, where the previously loosely stacked structure transforms into a tightly fused arrangement among the nanowires, indicating successful welding. The welding mechanism of AgNWs by NaCl treatment primarily occurs due to the replacement of PVP molecules by chloride ions, which have a smaller stereo-hindrance effect and stronger interactions with the AgNW surface [39]. The role of chloride ions was confirmed through infrared radiation (IR) and X-ray photoemission spectroscopy (XPS). In Fig. S9, after NaCl treatment, the characteristic stretching vibration peak of the carbonyl group at 1650 cm^−1^, observed in PVP-wrapped AgNWs, disappears. Additionally, Cl 2*p* spectra emerge with decreased peak signals in the C 1*s* spectrum, mainly originating from carbon atoms in the alkyl chain of PVP molecules. These results indicate the successful removal of insulating PVP molecules from the AgNW surfaces, consequently improving conductivity. In Fig. 3a, it is evident that the electron pathways of AgNWs are significantly increased by the combined effect of NaCl treatment and MXene coating. The MXene coating fills the large voids of insulation between AgNWs, further enhancing electrical conductivity.

Changes in the photoelectric properties of the AgNW film, welded AgNW film, and welded AgNW-MXene film with different layers were systematically explored. As depicted in Fig. 3b, an increase in the number of coating layers led to a decrease in transmittance for all three materials: the AgNW film, welded AgNW film, and welded AgNW-MXene film. Notably, the welded AgNW-MXene film exhibited the lowest transmittance among the three, primarily due to the presence of MXene nanosheets. It's worth highlighting that the NaCl welding process had no discernible effect on the optical transmittance of AgNW films. In fact, the welded AgNW film displayed a transmittance comparable to that of the AgNW film with the same number of coating layers. Regarding changes in sheet resistance with an increasing number of coating layers, Fig. 3c illustrates similar decreasing trends for the AgNW film, welded AgNW film, and welded AgNW-MXene film. Both NaCl welding and MXene coating contributed to the enhanced conductivity of the AgNW film. In order to more thoroughly evaluate the optoelectronic performance of FTEs, the Figure of Merit (FoM) was introduced and calculated using the equation: FoM = σ_dc_ / σ_opt_ = 188.5 / (R_s_ × (T^−1/2^–1)), where σ_dc_ and σ_opt_ represent the direct current conductivity and optical conductivity, respectively, R_s_ is the sheet resistance, T is the transmittance at a wavelength of 550 nm [40–42]. FoM takes into account both transmittance and sheet resistance, providing a comprehensive measure of FTE quality. As shown in Fig. 3d, the welded AgNW-MXene film achieved its highest FoM value of 563 and the corresponding electrical conductivity can reach 2.1 × 10^6^ S m^−1^ (Fig. S10) when the number of AgNW layers reached four. Notably, this level of FoM for the welded AgNW-MXene film surpasses the performance of other reported AgNW-based FTEs (Fig. S11), underscoring its outstanding optoelectronic capabilities.

The coating of MXene nanosheets has proven to be an effective strategy for preventing the oxidation of AgNW films in ambient air. As illustrated in Fig. 3e, the AgNW film experiences significant oxidation when exposed to room temperature air, while the welded AgNW-MXene film exhibits outstanding antioxidation properties, remaining stable for up to two months. Moreover, the AgNW-MXene film demonstrates impressive stability, withstanding temperatures as high as 200 °C and immersion in a harsh Na_2_S environment for 1 h without evident corrosion (Fig. 3f, g). These results underscore the superior thermal stability and anti-corrosion capabilities of the welded AgNW-MXene film compared to the welded AgNW film. Both the welded AgNW film and welded AgNW-MXene film also exhibit superior mechanical flexibility compared to traditional ITO film. This is effectively demonstrated through a bending deformation test, where the bending diameter ranged from 25 to 5 mm. As depicted in Fig. 3h, i, the relative sheet resistance variation (Δ*R*/*R*_0_) remains negligible for both the welded AgNW film and welded AgNW-MXene film, regardless of the bending direction. In contrast, the ITO film begins to show a sharp increase in Δ*R*/*R*_0_ when its bending diameter falls below 18 and 20 mm for inward and outward bending, respectively. This phenomenon can be explained by the SEM results presented in Fig. S12, which show an unchanged structure for the flexible AgNW film and a cracked structure for the fragile ITO film after bending deformations. Furthermore, a cyclic bending test was conducted to evaluate the mechanical durability of the ITO, welded AgNW, and welded AgNW-MXene films. In comparison to the ITO film, which exhibits a noticeable change in Δ*R*/*R*_0_ within the first few bending cycles, the welded AgNW and welded AgNW-MXene films maintain negligible changes in Δ*R*/*R*_0_, even after 10,000 bending cycles (Figs. 3j and S13). Moreover, tape-testing was performed to exhibit the affinity between MXene-AgNW and AgNW-PET. As shown in Fig. S14, when the tape-testing applied to the surface of MXene-AgNW and AgNW-PET film, the slight change in sheet resistance indicates the strong affinity between the materials. This observation could be attributed to the plasma treatment and salt welding between AgNWs, and electrostatic attraction between AgNWs and MXene nanosheets. The demonstrated strong affinity further supports the notable physical stability of the composite electrodes. These superior mechanical properties make the welded AgNW and welded AgNW-MXene films excellent choices for applications demanding consistent electrical performance under repeated mechanical deformations.

**Fig. 4:**
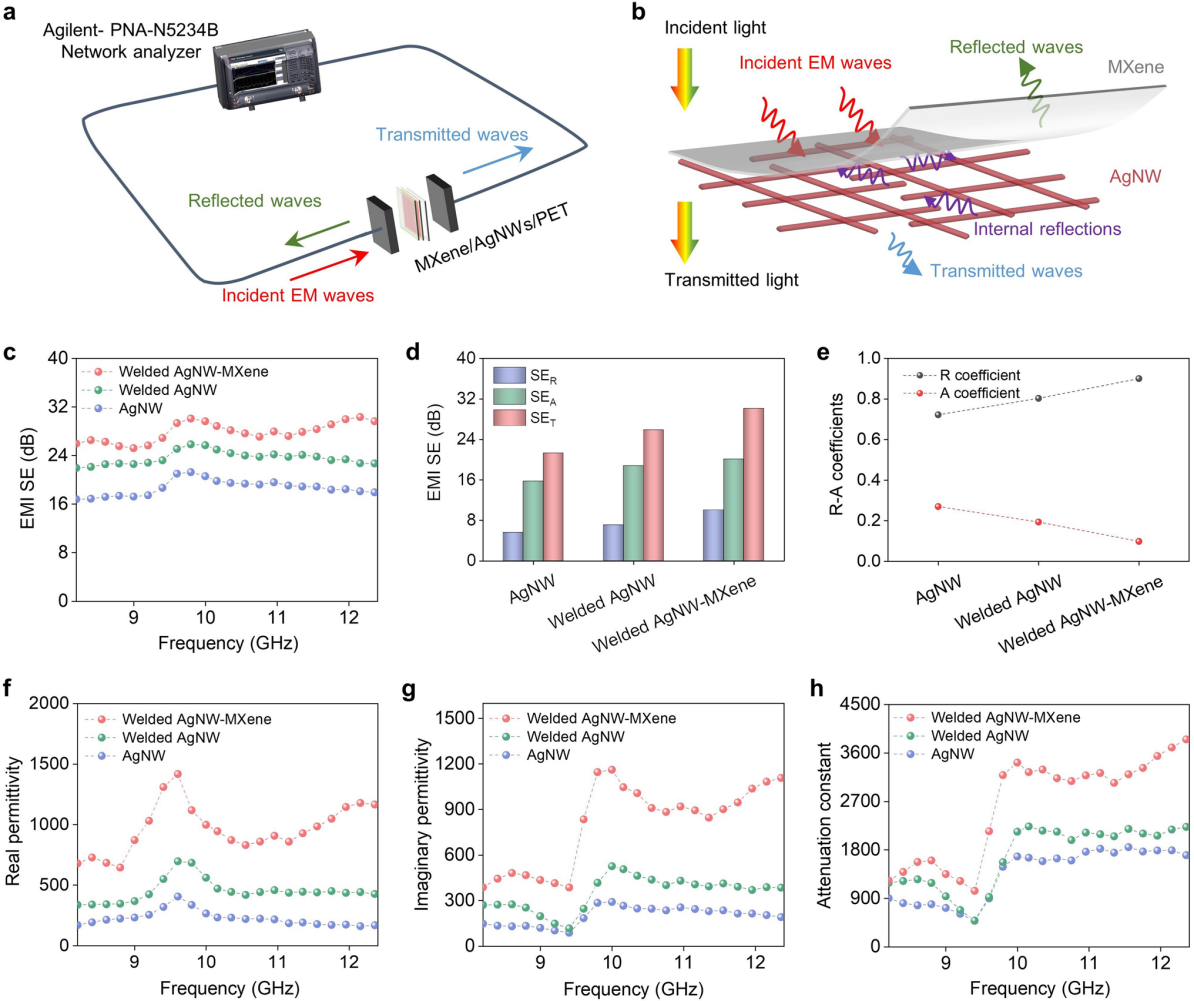
EMI shielding performance of the AgNW film, welded AgNW film and welded AgNW-MXene film. **a** Scheme of the EMI shielding test system. **b** Schematic diagram of EMI shielding mechanism of the welded AgNW-MXene film. **c** EMI SE in the frequency of 8.2–12.4 GHz, **d** average SER, SEA, SET values and **e** R, A coefficient of the AgNW film, welded AgNW film and welded AgNW-MXene film. Frequency dependence of **f** real and **g** imaginary part of relative complex permittivity, and **h** attenuation constant of the AgNW film, welded AgNW film and welded AgNW-MXene film

The welded AgNW-MXene film, characterized by outstanding optical and electrical properties, holds immense potential as a superior transparent EMI shielding material. To thoroughly investigate the EMI shielding performance and its underlying mechanism, we conducted a comparative study with the AgNW film and welded AgNW film as controls. Wherein, the AgNW, welded AgNW and welded AgNW-MXene films used in the EMI test have sheet resistances of 42.2, 10.4, and 9.4 Ω sq^−1^, respectively. Their corresponding transmittance values are about 95%, 96%, and 93%. As depicted in Fig. 4a, the EMI shielding test system involved placing the tested samples between two clamps to measure their performance. The specific EMI shielding mechanism of the welded AgNW-MXene film is illustrated in Fig. 4b. When incident electromagnetic (EM) waves strike the surface of the MXene layer, a portion of the EM waves is immediately reflected due to the impedance mismatch between air and the highly conductive MXene layer [29]. The remaining waves pass through the MXene layer with reduced energy and reach the surface of the AgNW network. Here, they generate multiple internal reflections due to the significant difference in electrical conductivity between AgNWs and Mxenes [43]. This results in ohmic losses and energy dissipation of the EM waves. Furthermore, the network structure of the AgNWs significantly enhances the EM wave absorbing ability by providing additional interfaces for multi-reflection and scattering of EM waves. Conductivity is a critical parameter for EMI SE, with higher conductivity translating to better EMI SE. As demonstrated in Fig. 4c, d, the welded AgNW-MXene film exhibits the highest total SE (*SE*_T_), absorption SE (*SE*_A_), and reflection SE (*SE*_R_) values compared to those of the AgNW film and welded AgNW film. These results align with the order of conductivity values for these films. Detailed changes in *SE*_T_, *SE*_A_, and *SE*_R_ values for these three films in the frequency range of 8.2 ~ 12.4 GHz can be found in Fig. S15a-c. The welded AgNW-MXene film showcases the best shielding performance, boasting an average EMI SE of 30.1 dB. This exceptional performance can be attributed to two key factors. Firstly, the coating of MXene nanosheets enhances the conductivity of AgNWs, contributing to improved EMI SE. Secondly, the synergistic effect between the network structure of AgNWs and the 2D layered structure of MXene nanosheets further enhances internal reflection and absorption of EMI waves, resulting in superior EMI SE.

The absorption coefficient (*A*) and reflection coefficient (*R*) are essential parameters that quantify a material's ability to absorb and reflect EM waves, respectively. These coefficients play a crucial role in accurately analyzing the EMI shielding mechanism [44]. In Fig. 4e, when compared to the AgNW film and welded AgNW film, the welded AgNW-MXene film exhibits the highest value for *R* and the lowest value for *A*. Figure S15d-f presents the corresponding changes in *R* and *A* values for these three films within the frequency range of 8.2 ~ 12.4 GHz. It is worth noting that, while the *A* is generally smaller than the *R* and the *SE*_R_ is smaller than the shielding effectiveness of *SE*_A_, *R* consistently exceeds *A* for all three films. This observation is explained by the fact that reflection precedes absorption in the EMI shielding process, with *SE*_A_ representing the film‘s ability to attenuate EM waves that manage to penetrate the material [45]. Consequently, the primary EM shielding mechanism for all three films is reflection.

The absorption capacity of EM waves by the welded AgNW-MXene film relies largely on the relative complex permittivity. Specifically, the real part (ε′) and the imaginary part (ε″) of the complex permittivity indicate the material's ability to store and dissipate electrical energies of EM waves, respectively. Figure 4f, g illustrates the ε′ and ε″ values for the AgNW film, welded AgNW film, and welded AgNW-MXene film within the frequency range of 8.2 ~ 12.4 GHz. Remarkably, the welded AgNW-MXene film exhibits the highest ε′ and ε″ values compared to the AgNW film and welded AgNW film. The increased ε′ can be attributed to dipole polarization induced by the functional groups present on MXene nanosheets and interfacial polarization occurring between MXene nanosheets and AgNWs. According to electronic theory, ε″ is inversely proportional to resistivity [46]. Thus, the coating of MXene nanosheets on the AgNW film results in the creation of more conductive pathways in the welded AgNW-MXene film, thereby increasing the ε″ value. The attenuation constant (α) is a critical parameter determining the attenuation capacity of a material for incoming EM waves. As demonstrated in Fig. 4h, the welded AgNW-MXene film boasts the highest α value among the three films. These findings collectively underscore that the EM wave shielding performance can be significantly enhanced by coating MXene nanosheets onto AgNW networks. In other words, the welded AgNW-MXene film outperforms the AgNW film and welded AgNW film, exhibiting superior EM shielding performance.

**Fig. 5:**
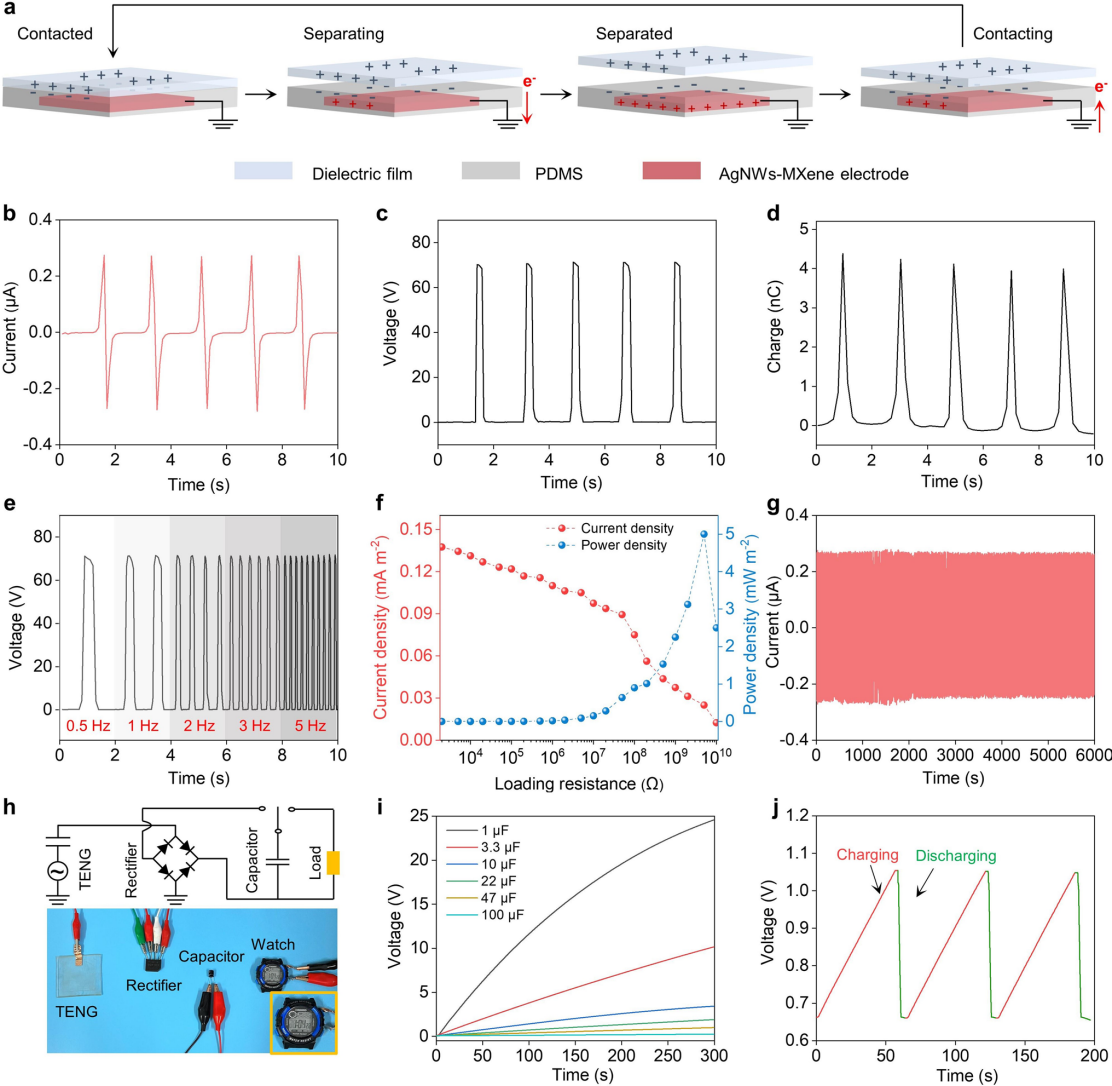
Applications of the AgNWs-MXene electrodes in TENG. **a** Schematic illustration of the working mechanism of AgNWs-MXene-based TENG in contact-separation mode. **b** Current, **c** voltage and **d** charge of the TENG. **e** Voltage of the TENG with different contact frequency. **f** Variation of the current density and power density with the diverse loading resistance. **g** Durability measurement of the TENG, where the voltage is recorded for 3000 cycles at a frequency of 0.5 Hz. **h** The equivalent circuit of a self-charging system (top) and a photograph of powering the electronic watch by the charged capacitor of 22 μF (bottom). **i** Charging capability curves of the capacitors from 1 to 100 μF under 0.5 Hz. **j** Voltage real-time charge/discharge profile of a 22 μF capacitor connected in the self-charging system

The AgNW-MXene electrode can also serve as the foundation for fabricating a flexible transparent TENG device. As depicted in Fig. S16, the AgNWs-MXene-based TENG (AM-TENG) was assembled by sandwiching a piece of AgNWs-MXene film between two layers of polydimethylsiloxane (PDMS). The operation of the AM-TENG relies on the synergy of triboelectrification and electrostatic induction within a contact-separation mode between the PDMS layer (frictional negative layer) and a dielectric film (movable frictional positive layer), as elaborated in Fig. 5a. Triboelectric charges are induced on the surfaces of the PDMS layer and dielectric film upon contact electrification. As these materials separate, the negative charges retained on the PDMS layer initiate the generation of positive charges on the AgNWs-MXene electrode layer. This process prompts free electrons to flow from the electrode layer to the ground, resulting in current and voltage generation-this is electrostatic induction. Electrostatic equilibrium is reached once the separation distance between the dielectric film and PDMS layer reaches its maximum. When the dielectric film once again approaches the PDMS layer, electrons flow from the ground back to the electrode layer, generating a reversed current and voltage. This cycle continues until the two layers come into contact once more. Throughout this repeated contact-separation process, the TENG continuously produces AC signals. Figure 5b-d displays typical electrical response signals, including current, voltage, and charge, generated by a 4 × 4c m^2^ AM-TENG when tapped by a stepping motor. Notably, the AM-TENG yields peak values of approximately 0.3 μA for current, 70 V for voltage, and 5 nC for charge. Furthermore, the voltage output signal of the AM-TENG was examined at different frequencies ranging from 0.5 to 5 Hz, as shown in Fig. 5e. The output voltage remains highly stable across various frequencies. By adjusting the external resistance from 2 kΩ to 10 GΩ, we assessed the variations in power density and current density, as presented in Fig. 5f. The power density, calculated using the formula PD = IR^2^/A, where PD represents power density, I is the output current, R is the load resistance, and A is the effective contact area, peaks at 5 mW m^−2^ with an external resistance of 5 GΩ. Conversely, current density shows a decline due to Ohmic loss [47]. To evaluate durability, the AM-TENG underwent testing at a "mild" frequency of 0.5 Hz. As demonstrated in Fig. 5g, even after more than 3000 cycles, the AM-TENG consistently generated a highly stable triboelectric output current, showcasing its remarkable robustness.

The electrical output performance of the AM-TENG proves highly effective for energy storage, particularly harnessed from human movements, which can be stored in capacitors to power various portable electronics. As illustrated in Fig. 5h and Movie S4, the AM-TENG efficiently harvests energy through hand tapping and stores it in a capacitor, facilitated by a rectifier bridge, to power devices such as electronic watches and time meters (Fig. S17a and Movie S5). When tapped by hand, the AM-TENG can effortlessly illuminate a series of 11 red LEDs (Fig. S17b and Movie S6). The charging voltage curves of the AM-TENG with different capacitors, ranging from 1.0 to 100 µF, are presented in Fig. 5i. These curves reveal that, at the same frequency, capacitors of 1.0 and 100 5µF reach 25 and 0.3 V within 300 s, respectively. To demonstrate the AM-TENG‘s effectiveness in powering electronic devices, Fig. 5j illustrates the charging and discharging processes of a watch. Initially, the voltage output from a 22 µF capacitor increases to 1.1 V in just 57 s, and subsequently returns nearly to its initial position after discharging. The capacitor is then recharged to 1.1 V as the AM-TENG is tapped again, ensuring continuous charging of the watch. Therefore, the AM-TENG developed in this study functions as a self-sustaining electronic system, capable of continuously charging various portable electronics. This innovation greatly enhances the utility and versatility of electronic devices, maximizing their potential value.

## Conclusions

In summary, we have demonstrated an advanced approach for fabricating highly stable FTEs with an ordered and homogeneous structure, achieved through the IL-enhanced assembly of NMs. The introduction of ILs plays a pivotal role in stabilizing the NM assembly by creating a stable IL-water interface, significantly reducing NM loss. This breakthrough enables the efficient and low-loss production of a 20 cm-wide roll of AgNW-MXene composite film, capable of stably lighting a blue LED. The resulting AgNW-MXene composite film exhibits exceptional properties, including a low sheet resistance of 9.4 Ω sq^−1^ and impressive optical transmittance of 93%. Moreover, the film's remarkable stability is evident as it endures exposure to various environments, such as extended periods in ambient air at room temperature, high temperatures up to 200 °C, and immersion in a Na_2_S solution for 1 h. The combination of excellent optoelectronic properties and environmental stability makes the AgNW-MXene composite film suitable for a wide range of applications. Notably, it demonstrates a remarkable EMI SE of 30.1 dB, surpassing the industry-standard requirement of 20 dB. Additionally, the composite film serves as the foundation for assembling a TENG device. This TENG device, powered by hand tapping, can effortlessly drive electronic watches or time meters in a self-charging system. Thus, our research unveils the immense potential of the composite films developed in this study, showcasing their suitability for diverse applications in the realm of flexible optoelectronics, thanks to their exceptional properties and stability.

## Supplementary Information

Below is the link to the electronic supplementary material.

Supplementary file1 (PDF 1037 KB)

Supplementary file2 (MP4 13368 kb)

Supplementary file3 (MP4 11935 kb)

Supplementary file4 (MOV 78684 kb)

Supplementary file5 (MP4 1162 kb)

Supplementary file6 (MP4 882 kb)

Supplementary file7 (MP4 2998 kb)
