# Supplementary material for: Ionic Liquid-Enhanced Assembly of Nanomaterials for Highly Stable Flexible Transparent Electrodes
Source: Nanomicro Lett. 2024 Mar 4;16:140. doi: 10.1007/s40820-024-01333-4 (PMC10912071; doi:10.1007/s40820-024-01333-4)
Supplement: Supplementary file 1 — Supplementary file1 (PDF 1037 KB) [file 40820_2024_1333_MOESM1_ESM.pdf]

Supporting Information for

Ionic Liquid-Enhanced Assembly of Nanomaterials for Highly Stable Flexible Transparent Electrodes

Jianmin Yang[1,2], Li Chang[3], Xiqi Zhang[1,4], Ziquan Cao[1,]*, Lei Jiang[1,4,]*

[1]Key Laboratory of Bio-inspired Materials and Interfacial Science, Technical Institute of Physics and Chemistry, Chinese Academy of Sciences, Beijing 100190, P. R. China

[2]University of Chinese Academy of Sciences, Beijing 100049, P. R. China

[3]College of Chemistry and Chemical Engineering, Chongqing University of Technology, Chongqing 400054, P. R. China

[4]Binzhou Institute of Technology, Binzhou 256600, P. R. China

*Corresponding authors. E-mail: caoziquan@mail.ipc.ac.cn (Ziquan Cao); jianglei@iccas.ac.cn (Lei Jiang)

Supplementary Figures and Tables

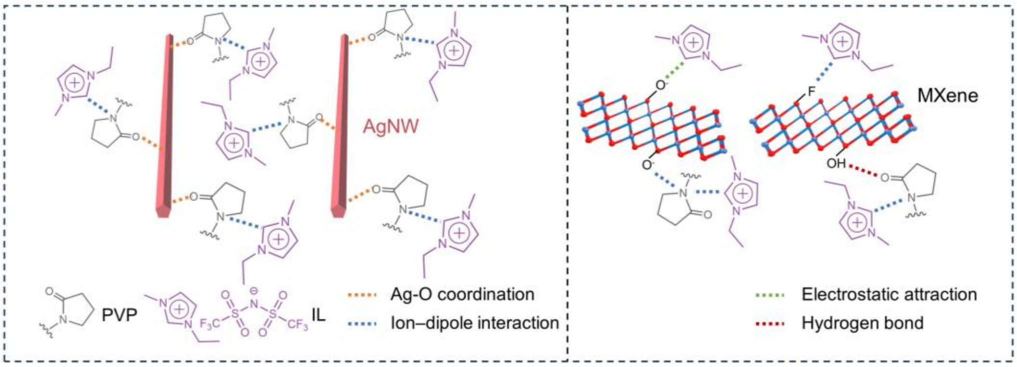

Fig. S1 Schematic illustration of the mechanism of IL-enhanced assembly of NMs (AgNWs and MXene)

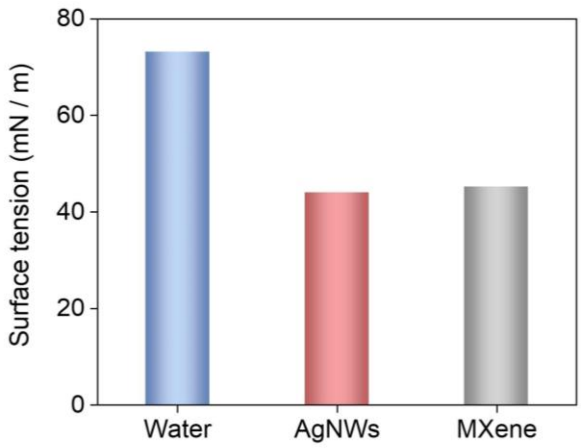

Fig. S2 The surface tensions of the water, AgNWs and MXene

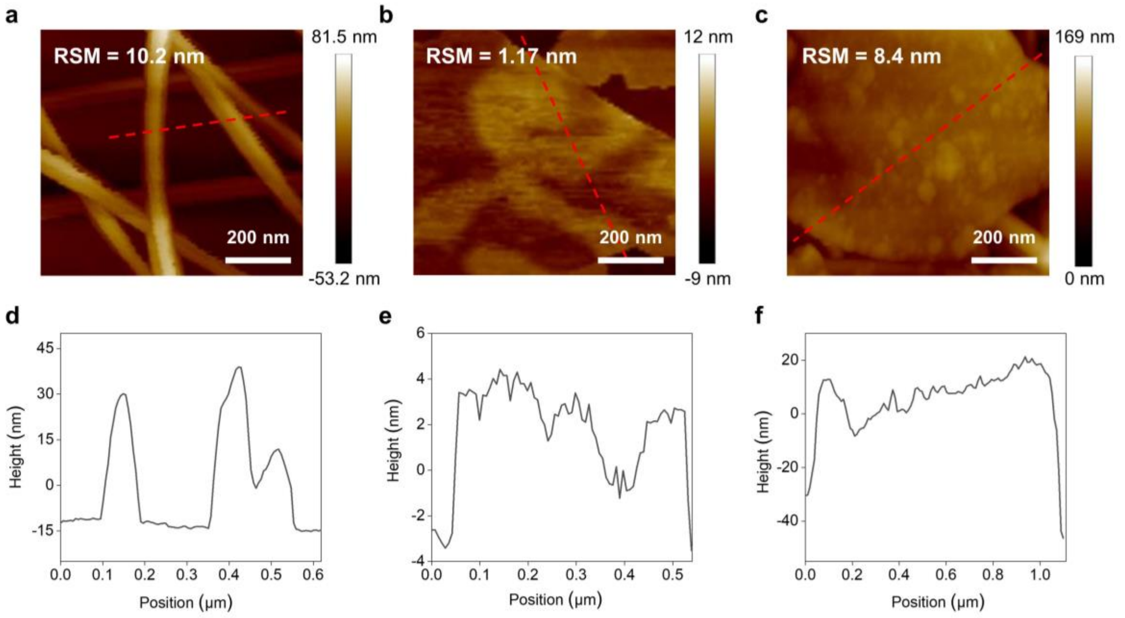

Fig. S3 AFM images of the AgNWs, MXene, and AgNWs-MXene films **a-c**, and the crresponding hight profiles **d-f**

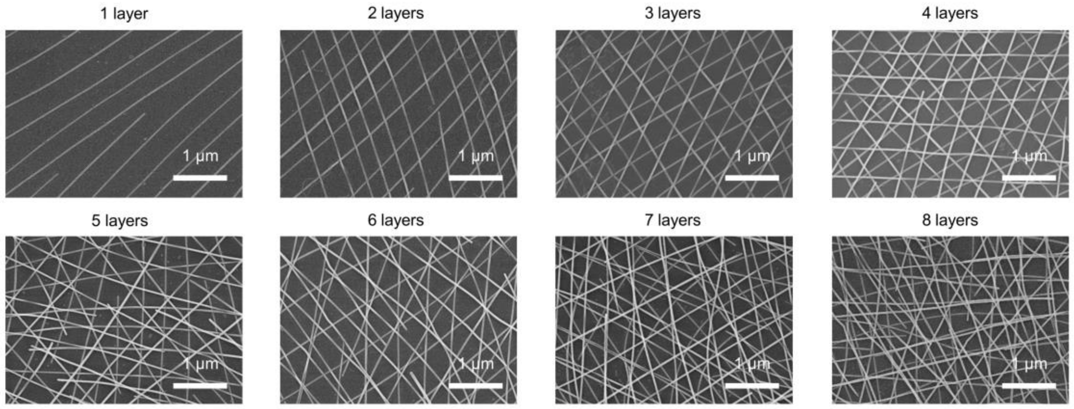

Fig. S4 SEM images of the ordered AgNW networks with different layers

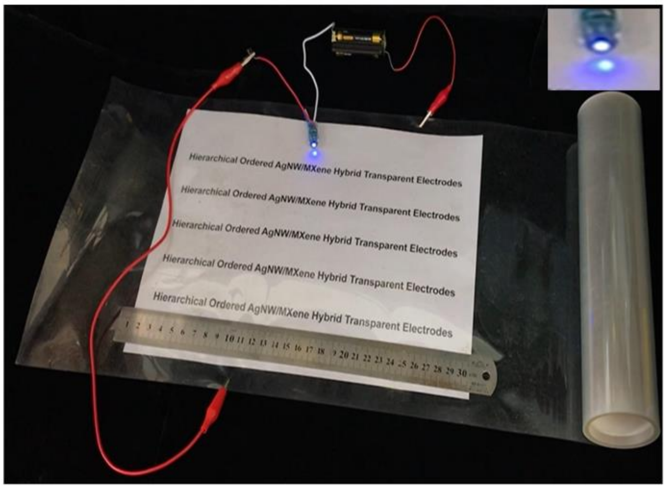

Fig. S5 A photograph of a 20 cm-wide roll of transparent AgNW-MXene electrode on PET substrate, which can light a blue LED lamp stably

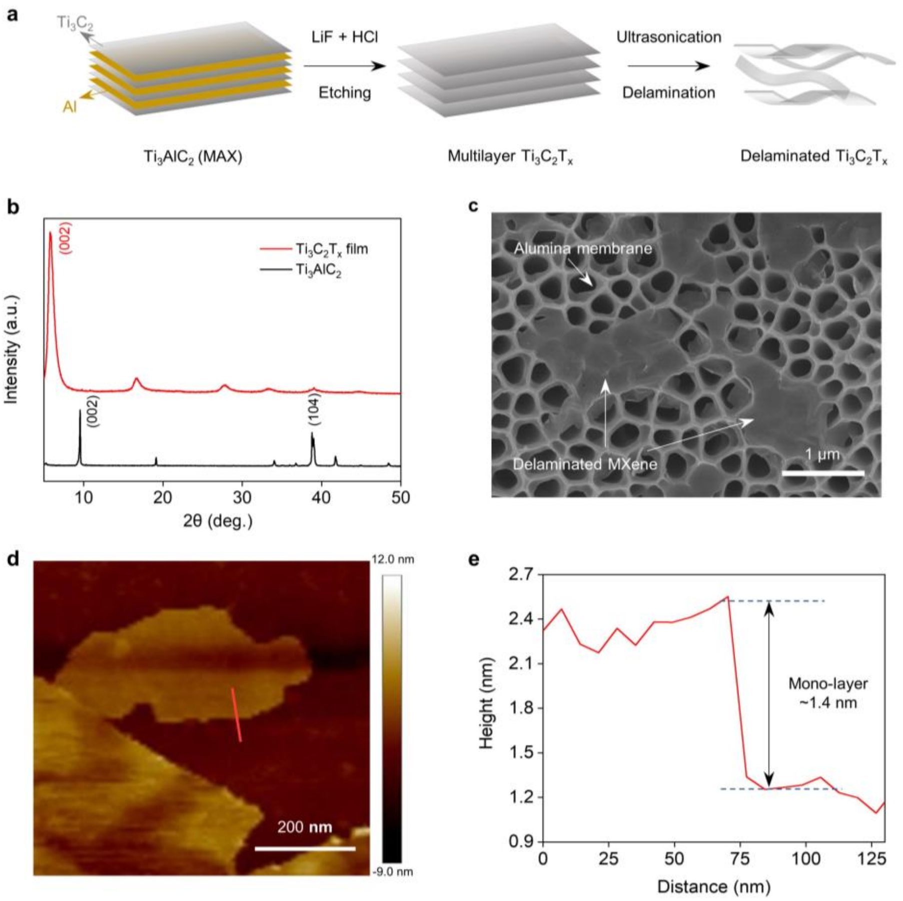

Fig. S6 a Schematic of the fabrication process of the delaminated MXene-$Ti_3C_2T_x$. **b** XRD patterns of the raw Ti_3AlC_2 and delaminated $Ti_3C_2T_x$ film. **c** SEM image of the prepared MXene nanosheet with the diameter of 1-2 μm. **d, e** AFM image and AFM section analysis of the prepared MXene nanosheet

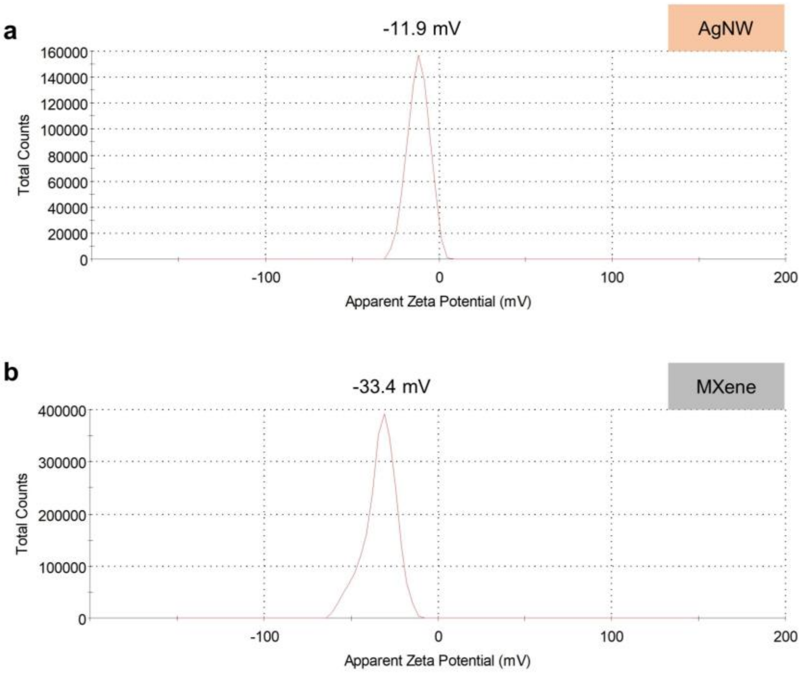

Fig. S7 Zeta-potentials of **a** the AgNW and **b** the $Ti_3C_2T_x$ MXene nanosheets

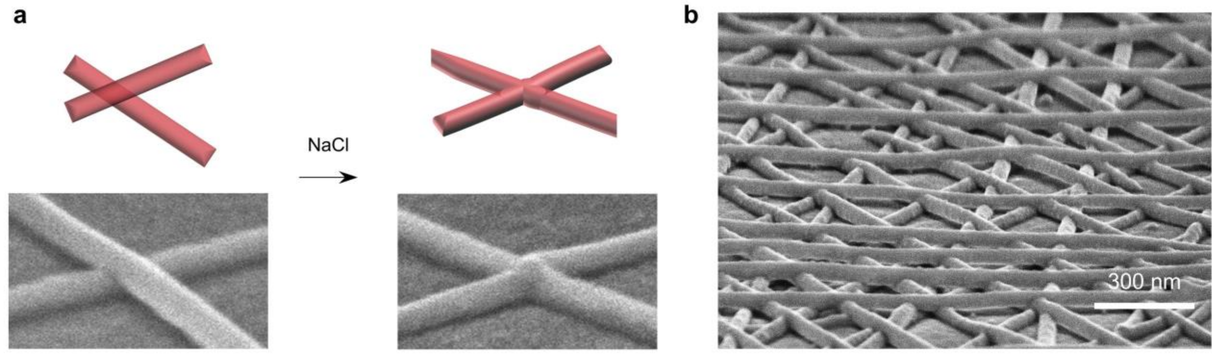

Fig. S8 a Welding of AgNWs network by NaCl solution treatment. **b** Tilted cross-sectional SEM image of the welded AgNW film with four layers

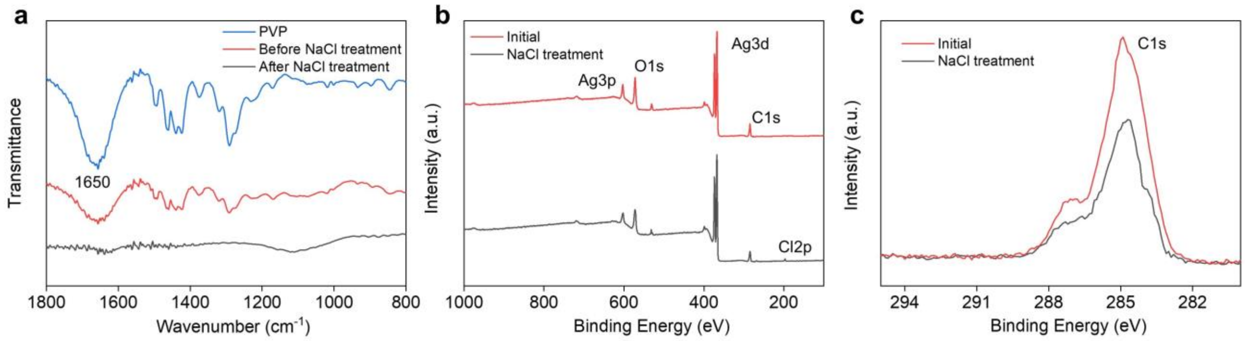

Fig. S9 a IR spectras of PVP and AgNW film before and after NaCl treatment. XPS survey **b** and C1s spectrum **c** of the AgNW film in initial state and after NaCl treatment

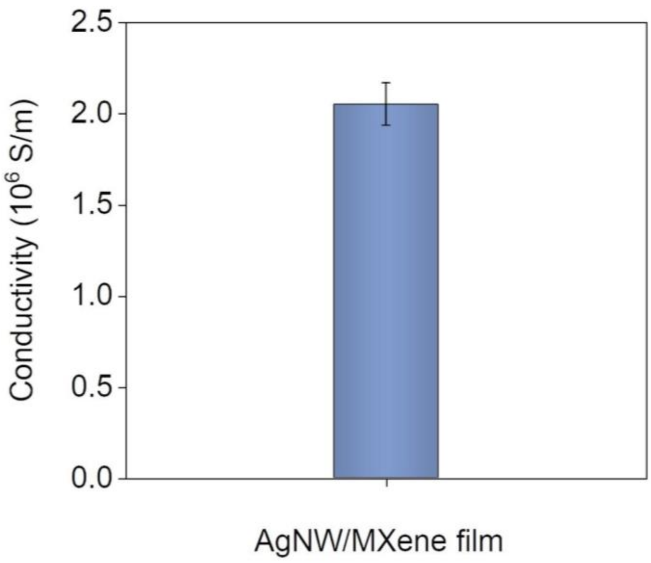

Fig. S10 The electrical conductivity of the AgNW/MXene composite electrode with four layers of AgNW

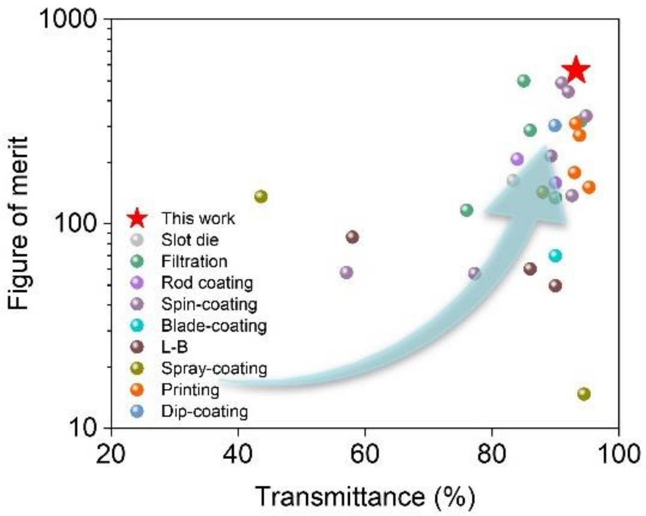

Fig. S11 Transmittance versus FoM for our AgNW-MXene FTEs with previously reported AgNW-based FTEs prepared by different methods (slot die, filtration, rod coating, spin-coating, blade-coating, L-B, spray-coating, printing and dip-coating) for comparison [S1-S28]

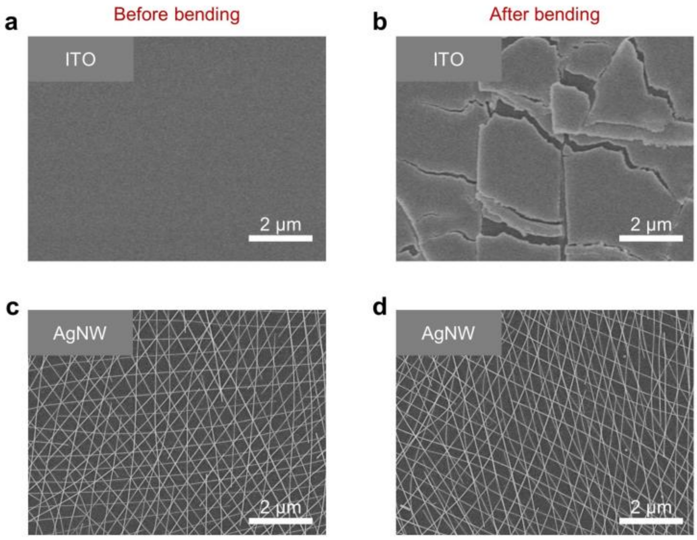

Fig. S12 SEM images of the ITO film **a, b** and AgNW film **c, d** before and after bending deformations

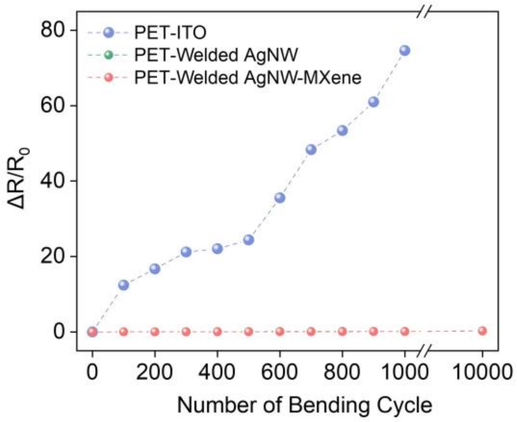

Fig. S13 Variation in ΔR/R0 versus the number of bending inward cycles from a diameter of 25 mm to 5 mm for the ITO, welded AgNW and welded AgNW-MXene film

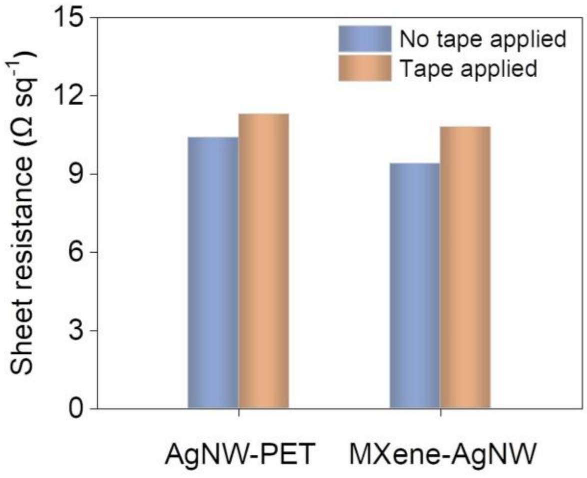

Fig. S14 The effect of the scotch tape test on the sheet resistance of the AgNW/PET and MXene/AgNW films

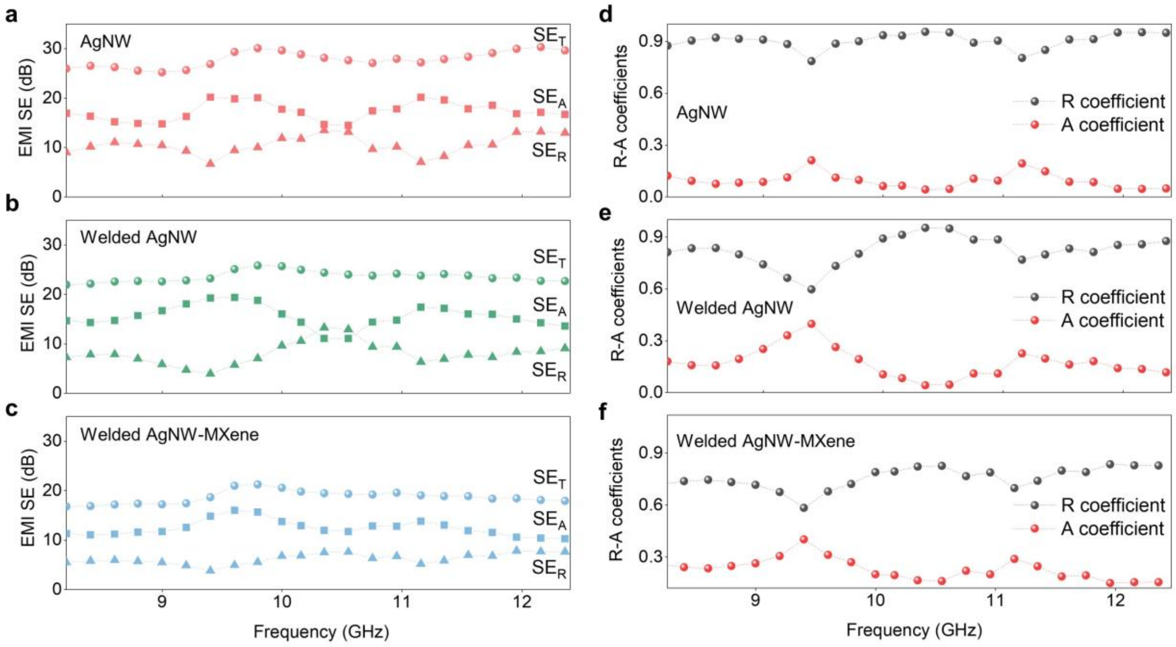

Fig. S15 Frequency dependence of the SER, SEA, SET values **a-c** and R-A coefficient **d-f** for the AgNW film, welded AgNW film and the welded AgNW-MXene film

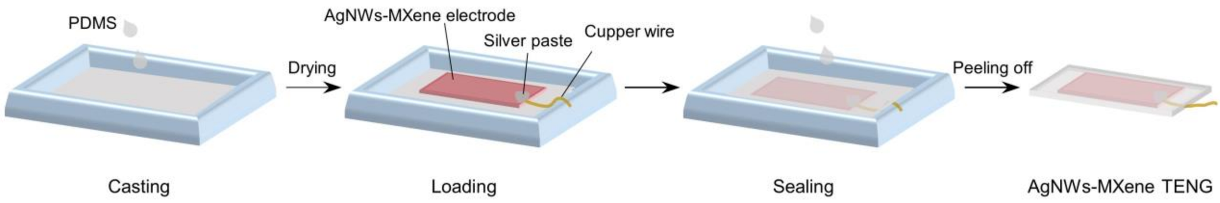

Fig. S16 Schematic illustration of fabrication process of AgNWs-MXene-based TENG

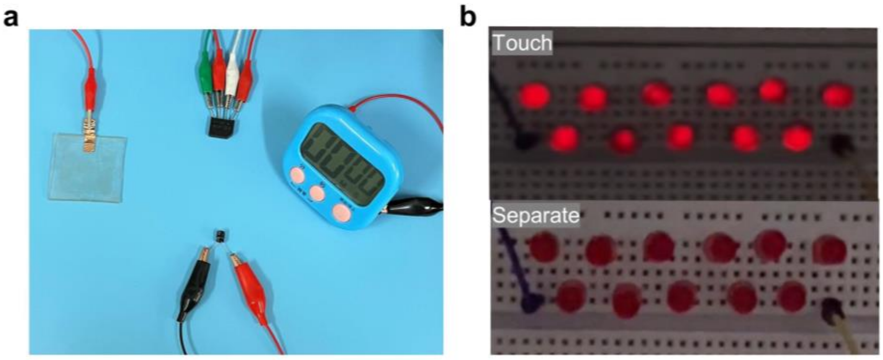

Fig. S17 Images of a time meter **a** and 11 red LEDs **b** powered by tapping the AM-TENG

Supplementary References

[S1] J. K. Han, J. K. Yang, W. W. Gao, H. Bai, Ice-templated, large-area silver nanowire pattern for flexible transparent electrode. Adv. Funct. Mater. **31**(16), (2021). https://doi.org/10.1002/adfm.202010155

[S2] J. L. Wang, Y. R. Lu, H. H. Li, J. W. Liu, S. H. Yu, Large area co-assembly of nanowires for flexible transparent smart windows. J. Am. Chem. Soc. **139**(29), 9921-9926 (2017). https://doi.org/10.1021/jacs.7b03227

[S3] S. K. Duan, Q. L. Niu, J. F. Wei, J. B. He, Y. A. Yin et al., Water-bath assisted convective assembly of aligned silver nanowire films for transparent electrodes. Phys. Chem. Chem. Phys. **17**(12), 8106-8112 (2015). https://doi.org/10.1016/j.cej.2023.144065

[S4] T. Kim, A. Canlier, G. H. Kim, J. Choi, M. Park et al., Electrostatic spray deposition of highly transparent silver nanowire electrode on flexible substrate. ACS Appl. Mater. Interfaces **5**(3), 788-794 (2013). https://doi.org/10.1021/am3023543

[S5] Y. R. Jang, W. H. Chung, Y. T. Hwang, H. J. Hwang, S. H. Kim et al., Selective wavelength plasmonic flash light welding of silver nanowires for transparent electrodes with high conductivity. ACS Appl. Mater. Interfaces **10**(28), 24099-24107 (2018). https://doi.org/10.1021/acsami.8b03917

[S6] J. Lee, J. Y. Woo, J. T. Kim, B. Y. Lee, C. S. Han, Synergistically enhanced stability of highly flexible silver nanowire/carbon nanotube hybrid transparent electrodes by plasmonic welding. ACS Appl. Mater. Interfaces **6**(14), 10974-10980 (2014). https://doi.org/10.1021/am502639n

[S7] H. H. Tang, H. R. Feng, H. K. Wang, X. J. Wang, J. J. Liang et al., Highly conducting MXene-silver nanowire transparent electrodes for flexible organic solar cells. ACS Appl. Mater. Interfaces **11**(28), 25330-25337 (2019). https://doi.org/10.1021/acsami.9b04113

[S8] S. De, T. M. Higgins, P. E. Lyons, E. M. Doherty, P. N. Nirmalraj et al., Silver nanowire networks as flexible, transparent, conducting films: Extremely high DC to optical conductivity ratios. ACS Nano **3**(7), 1767-1774 (2009). https://doi.org/10.1021/nn900348c

[S9] A. Kim, Y. Won, K. Woo, C. H. Kim, J. Moon, Highly transparent low resistance ZnO/Ag nanowire/ZnO composite electrode for thin film solar cells. ACS Nano **7**(2), 1081-1091 (2013). https://doi.org/10.1021/nn305491x

[S10] W. Gaynor, S. Hofmann, M. G. Christoforo, C. Sachse, S. Mehra et al., Color in the corners: ITO-free white OLEDs with angular color stability. Adv. Mater. **25**(29), 4006-4013 (2013). https://doi.org/10.1002/adma.201300923

[S11] J. A. Spechler, T.-W. Koh, J. T. Herb, B. P. Rand, C. B. Arnold, A transparent, smooth, thermally robust, conductive polyimide for flexible electronics. Adv. Funct. Mater. **25**(48), 7428-7434 (2015). https://doi.org/10.1002/adfm.201503342

[S12] Z. Jiang, K. Fukuda, X. M. Xu, S. Park, D. Inoue et al., Reverse-offset printed ultrathin Ag mesh for robust conformal transparent electrodes for high-performance organic photovoltaics. Adv. Mater. **30**(26), 7 (2018). https://doi.org/10.1002/adma.201707526

[S13] J. H. Seo, I. Hwang, H. D. Um, S. Lee, K. Lee et al., Cold isostatic-pressured silver nanowire electrodes for flexible organic solar cells via room-temperature processes. Adv. Mater. **29**(30), 1701479 (2017). https://doi.org/10.1002/adma.201701479

[S14] W. W. Xiong, H. L. Liu, Y. Z. Chen, M. L. Zheng, Y. Y. Zhao et al., Highly conductive, air-stable silver nanowire@iongel composite films toward flexible transparent electrodes. Adv. Mater. **28**(33), 7167-7172 (2016). https://doi.org/10.1002/adma.201600358

[S15] J. W. Liu, J. L. Wang, Z. H. Wang, W. R. Huang, S. H. Yu, Manipulating nanowire assembly for flexible transparent electrodes. Angew. Chem. Int. Ed. **53**(49), 13477-13482 (2014). https://doi.org/10.1002/anie.201408298

[S16] H. Yu, Y. Tian, M. Dirican, D. J. Fang, C. Y. Yan et al., Flexible, transparent and tough silver nanowire/nanocellulose electrodes for flexible touch screen panels. Carbohydr. Polym. **273**, 118539 (2021). https://doi.org/10.1016/j.carbpol.2021.118539

[S17] L. L. Meng, M. Zhang, H. H. Deng, B. J. Xu, H. Q. Wang et al., Direct-writing large-area cross-aligned Ag nanowires network: Toward high-performance transparent quantum dot light-emitting diodes. CCS Chem. **3**(8), 187-195 (2021). https://doi.org/10.31635/ccschem.020.202000402

[S18] N. M. Nair, M. M. Jahanara, D. Ray, P. Swaminathan, Photoresponse of a printed transparent silver nanowire-zinc oxide nanocomposite. Flex. Print. Electron. **6**(4), 045004 (2021). https://doi.org/10.1088/2058-8585/ac2e48

[S19] S. Y. Huang, Q. Z. Zhang, F. Yang, D. T. Gangadharan, P. D. Li et al., A facile way for scalable fabrication of silver nanowire network electrodes for high-performance and foldable smart windows. J. Mater. Chem. A **8**(17), 8620-8628 (2020). https://doi.org/10.1039/c9ta14030a

[S20] Y. J. Ge, X. D. Duan, M. Zhang, L. Mei, J. W. Hu et al., Direct room temperature welding and chemical protection of silver nanowire thin films for high performance transparent conductors. J. Am. Chem. Soc. **140**(1), 193-199 (2018). https://doi.org/10.1021/jacs.7b07851

[S21] B. Deng, P. C. Hsu, G. C. Chen, B. N. Chandrashekar, L. Liao et al., Roll-to-roll encapsulation of metal nanowires between graphene and plastic substrate for high-performance flexible transparent electrodes. Nano Lett. **15**(6), 4206-4213 (2015). https://doi.org/10.1021/acs.nanolett.5b01531

[S22] P. C. Wang, C. Zhang, M. J. Q. Wu, J. H. Zhang, X. Ling et al., Scalable solution-processed fabrication approach for high-performance silver nanowire/MXene hybrid transparent conductive films. Nanomaterials **11**(6), 12 (2021). https://doi.org/10.3390/nano11061360

[S23] Y. Yang, J. L. Wang, L. Liu, Z. H. Wang, J. W. Liu et al., A room-temperature environmentally friendly solution process to assemble silver nanowire architectures for flexible transparent electrodes. Nanoscale **9**(1), 52-55 (2017). https://doi.org/10.1039/c6nr06984c

[S24] Y. N. Sun, M. J. Chang, L. X. Meng, X. J. Wan, H. H. Gao et al., Flexible organic photovoltaics based on water-processed silver nanowire electrodes. Nat. Electron. **2**(11), 513-520 (2019). https://doi.org/10.1038/s41928-019-0315-1

[S25] H. M. Sim, H. K. Kim, Highly flexible Ag nanowire network covered by a graphene oxide nanosheet for high-performance flexible electronics and anti-bacterial applications. Sci. Technol. Adv. Mater. **22**(1), 794-807 (2021). https://doi.org/10.1080/14686996.2021.1963640

[S26] A. J. Stapleton, R. A. Afre, A. V. Ellis, J. G. Shapter, G. G. Andersson et al., Highly conductive interwoven carbon nanotube and silver nanowire transparent electrodes. Sci. Technol. Adv. Mater. **14**(3), 8 (2013). https://doi.org/10.1088/1468-6996/14/3/035004

[S27] J. Y. Song, J. H. Oh, D. Choi, S. M. Park, Highly efficient patterning technique for silver nanowire electrodes by electrospray deposition and its application to self-powered triboelectric tactile sensor. Sci. Rep. **11**(1), 21437 (2021). https://doi.org/10.1038/s41598-021-01043-6

[S28] J. Lee, I. Lee, T. S. Kim, J. Y. Lee, Efficient welding of silver nanowire networks without post-processing. Small **9**(17), 2887-2894 (2013). https://doi.org/10.1002/smll.201203142
